# Edaravone Decreases Paraquat Toxicity in A549 Cells and Lung Isolated Mitochondria

**Published:** 2014

**Authors:** Mohammad Shokrzadeh, Fatemeh Shaki, Ebrahim Mohammadi, Neda Rezagholizadeh, Fatemeh Ebrahimi

**Affiliations:** a *Pharmaceutical Sciences Research Center, Mazandaran University of Medical Sciences, Sari, Iran*; b *Department of Toxicology and Pharmacology, Faculty of Pharmacy, Mazandaran University of Medical Sciences, Sari, Iran.*; c *Kurdistan Environmental Health Research Center, Kurdistan University of Medical Sciences, Sanandaj, Iran.*

**Keywords:** Edaravone, Paraquat, Oxidative stress, Lung mitochondria, A549 cells

## Abstract

Edaravone, an antioxidant and radical scavenger, showed protective effects against oxidative stress-like condition. Paraquat (PQ) is toxic herbicide considerable evidence suggests that oxidative stress and mitochondrial dysfunction contribute to PQ toxicity. In this study, protective effect of edaravone against PQ induced toxicity and reactive oxygen species (ROS) generation in A549 cells and lung isolated mitochondria were evaluated.

A549 cells and lung isolated mitochondria were divided into control group, PQ group, edaravone group and PQ plus edaravone-pretreated group. Cellular and mitochondrial viability assayed using MTT test and ROS generations in both cellular and mitochondrial fraction were determined by fluorometry using DCFH-DA as indicator.

Our results showed that edaravone (5–100 µM) prevented PQ (500 µM) induced cytotoxicity in A549 cells that the best protective effect was observed at concentration of 50 µM of edaravone. In addition, PQ-induced ROS generation in A549 cells significantly inhibited by edaravone. Moreover, PQ decreased mitochondria viability and also increased ROS generation in lung isolated mitochondria that edaravone (25–400 µM) markedly inhibited these toxic effects.

In overall, the results of this study suggest that lung mitochondria maintenance is essential for maintaining PQt cytotoxicity and Edaravone is a protective drug against PQ toxicity *in-vitro.*

## Introduction

Edaravone (3-methyl-1- phenyl-2-pyrazolin-5-one), a potent and novel scavenger of free radicals, not only is highly efficient in quenching the hydroxyl radicals but also prevents oxidative stress-induced cellular damage (1). Of several mechanisms suggested for protective effect of edaravone, reactive oxygen species (ROS) *scavenging *is the most acceptable mechanism leading to protection of various cells such as endothelial cells, neurons and myocardial cells, against damage by oxidative stress condition (2). Recently, several studies showed that edaravone could improve mitochondrial function and protect mitochondrial oxidative damage (3-5).

Paraquat (PQ) is one of the most widely used herbicides that cause severe toxicity in humans and animals (6). PQ poisoning is a common cause of mortality in presentations of poisoning in many developing countries and accounts for up to a third of all suicides worldwide poisoning has been observed in patients who ingest the pesticide either accidentally or intentionally as a suicide attempt (7).In acute PQ poisoning, patients develop acute renal failure, acute lung injury and progressive pulmonary fibrosis and death occurs as a result of respiratory failure (8).The mechanisms of paraquat toxicity are related to the production of the superoxide anion, which can lead to the generation of more toxic reactive oxygen species (ROS) and cell damage (9).Oxidative stress may be defined as an alteration in the steady-state balance between oxidant and antioxidant agents in the cells (10, 11). Mitochondria are presumed to be the major source of oxidative stress and free radicals in cells and are also determinants in cell’s death (12).In fact, PQ is a prototypic compound is known to exert its toxic effects via oxidative stress and mitochondria are considered the most important targets of paraquat in animal tissues (6).

Due to lack of effective antidote in PQ poisoning and antioxidant and free radical scavenging activities of edaravone, we planned to investigate the protective effect of edaravone on paraquat induced mitochondrial toxicity in the PC1_2_ cell line and rat isolated lung mitochondria subjected to paraquat.

## Experimental

*Chemicals*

All chemicals used were of the highest quality and purchased from Sigma Chemical Co. (St. Louis, MO, USA). Organic solvents of analytical grade, HPLC grade or the best pharmaceutical grade were used.

*Cells culture and groups*

Human type II alveolar epithelial cells (A549 cells) were grown in endothelial cell growth medium (RPMI 1640) supplemented with 10% heat inactivated fetal bovine serum at 37 ^°^C in a humid atmosphere of 5% CO2. The medium was changed every other day and cells were used at 80–90% confluency. A549 cells were incubated with different concentrations of PQ (100, 250, 500, 1000 and 2000 µM) at 37 ^◦^C for 24 h and then PQ LC50 was determined. ThenA549 cells were divided into four groups including control (C), PQ (P), edaravone-treated (E), and PQ plus edaravone (P+Q) groups. The cells were exposed to PQ LC50 (500µM), and edaravone with 50µmol/L concentration were used in experiments.

*Determination of reactive oxygen species (ROS)* *in A549 cells*

The A549 cells were pre-incubated for 1 h with and without 100 µM edaravone then PQ (500 µM) was added to group P and P+E and incubated for 12, 24 and 48 h. ROS formation was determined with DCFH-DA (final concentration 20 µM) and the fluorescence intensity of DCF was measured using a Shimadzu RF5000U fluorescence spectrophotometer. Excitation and emission wavelengths were 500 and 520 nm, respectively. The results were expressed as fluorescent intensity per 10^6^ cells (13).

*Isolation of mitochondria*

Mitochondria were isolated from fresh rat’s lung and all experiments were conducted according to the ethical standards and protocols approved by the Committee of Animal Experimentation of Mazandaran University of Medical Sciences, Sari, Iran. The animals were decapitated and their lungs were dissected out quickly; the removed lungs were rinsed rapidly using isotonic saline buffer. The lungs were minced in a cold manitol solution containing 0.225 M D-mannitol, 75 mM sucrose, and 0.2 mM ethylenediaminetetraacetic acid (EDTA). The minced lungs were gently homogenized in a glass homogenizer with a Teflon pestle and then centrifuged at 1000 × g for 10 min at 4 ^◦^C to remove the nuclei, unbroken cells, and other non-sub cellular tissue. The supernatants were centrifuged at 10,000 × g for 10 min twice and the mitochondrial sediments were suspended in Tris solution containing 0.05 M Tris-HCl buffer (pH 7.4), 0.25 M sucrose, 20 mM KCl, 2.0 mM MgCl_2_, and 1.0 mM Na_2_HPO_4_ at 4 ^◦^C before assay (14).Protein concentrations were determined by the Coomassie blue protein-binding method using BSA as the standard (15). Mitochondria were prepared fresh for each experiment and used within 4 h of isolation. All the above mentioned steps operated strictly on ice to guarantee the isolation of high-quality mitochondrial preparation.

*Assessment of mitochondrial toxicity*

Mitochondrial toxicity was assessed by measuring the reduction of MTT (3-[4,5-dimethylthiazol-2-yl]-2,5-diphenyltetrazolium bromide with minor modification of Ghazi-Khansari *et al.* (9).

*Determination of EC50 for PQ in isolated lung mitochondria*

To avoid either non-toxic or very toxic conditions in this study, we determined EC_50_ concentrations for PQby MTT assay and this concentration was used in all experiments. 

*Determination of ROS* *in isolated lung mitochondria*

The mitochondrial ROS measurement was measured flourometrically using DCFH-DA. Briefly, isolated lung mitochondria were treatedaccording to the individual experimentin respiration buffer containing (0.32 mM sucrose,10 mM Tris, 20 mM Mops, 50 μM EGTA, 0.5 mM MgCl_2_, 0.1 mM KH_2_PO_4_and 5 mM sodium succinate) (16). In the interval times of 5, 15, 30, 45 and 60 min. following the PQ addition, a sample was taken and DCFH-DA was added (final concentration, 10 μM) to mitochondria which was then incubated for 10 min. The amount of ROS generation in isolated lung mitochondria was determined through a Shimadzu RF5000U fluorescence spectrophotometer at 485-nm excitation and 520-nm emission wavelength. The results were expressed as fluorescent intensity per 1mg protein mitochondria (16).

## Results

**Figure 1:**
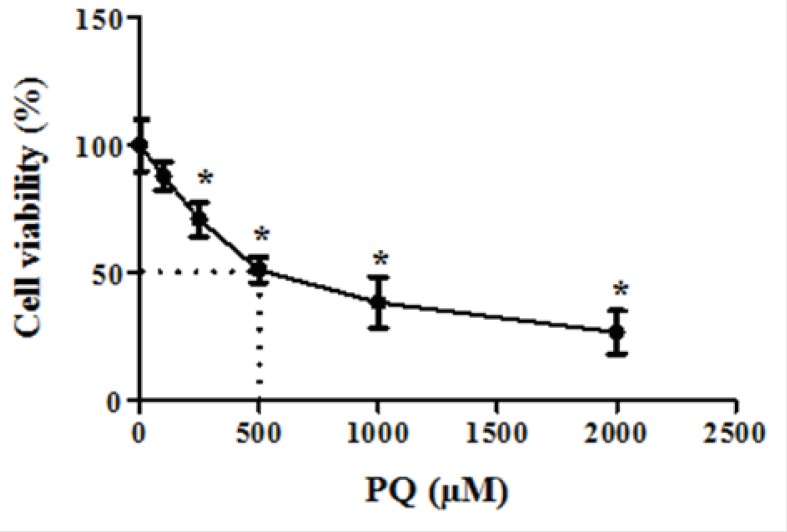
The dose-response effect of parquet on cell viability

**Figure 2:**
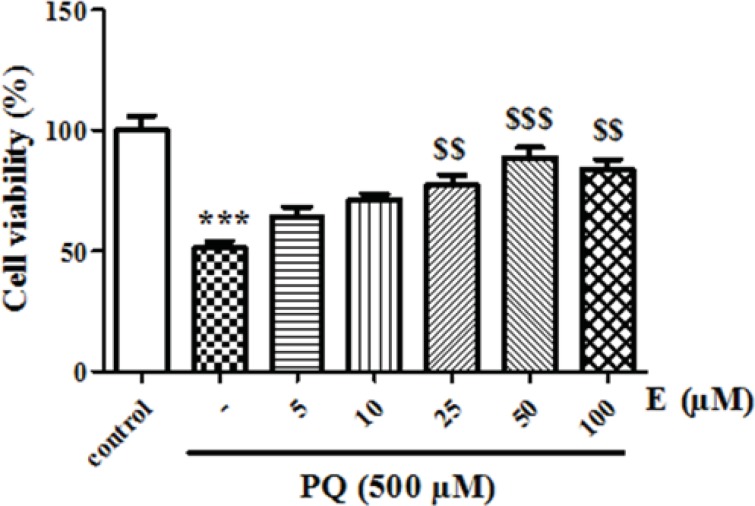
The effect of edaravone on cell viability

First, we determined LC_50_ of PQ on human lung epithelial A549 cell after 24 h incubation. As shown in Figure 1, human lung epithelial A549 cells were exposed to different concentrations PQ (0-2000 μM) for 24 h and cytotoxicity was determined using the MTT test. PQ up to the concentration of 100 μM, did not produce significant cytotoxicity, although when PQ concentration increased to 250 μM, cytotoxicity was observed in a concentration dependent manner. In MTT assay cell viability was significantly (p<0.05) decreased to 71%, 50%, 38% and 27% for the concentrations of 250, 500, 1000 and 2000 μM respectively (Figure 1).The LC_50_ of PQ is defined as the concentration which decreases cell viability down to 50% following a 24 h incubation, a cell viability decline to 50% was observed at concentration of 500 µM. Then, we examined the best protective concentration of edaravone against cytotoxicity induced by PQ in human lung epithelial A549 cells. As seen in Figure 2, edaravone pretreatment (5–100 µM) significantly protected cells from the toxicity induced by PQ (500 µM). Maximal cytoprotective effect was observed at 50 µM of edaravone (p<0.05). Given this result, 50 µM edaravone was chosen in subsequent cellular experiments. 

Human lung epithelial A549 cells (10^6^ cells/mL) were incubated at 37 ^◦^C for 24 h following the addition of different concentration of PQ (0, 100, 250, 500, 1000 and 2000 μM), cytotoxicity was assessed using MTT assays as described in methods and materials. Data represent the mean±S.D. of six separate experiments.

*P<0.05; compared with control group.

Human lung epithelial A549 cells (10^6^ cells/mL) were incubated at 37 ^◦^C with 5–100 µM edaravone for 1 h and followed by exposure to 500 µM PQ for 24 h. Cell viability was assessed using MTT assays as described in methods and materials. Data represent the mean±S.D. of six separate experiments.

***P<0.001; compared with control group.

^$$^ *P*<0.01; compared with PQ group.

^$$$^ *P*<0.001; compared with PQ group.

**Figure 3:**
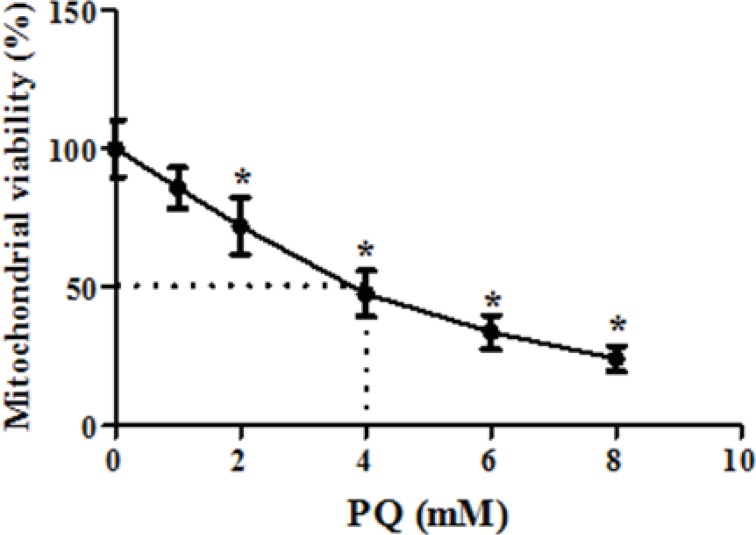
The dose-response effect of parquet on isolated lung mitochondria viability

**Figure 4:**
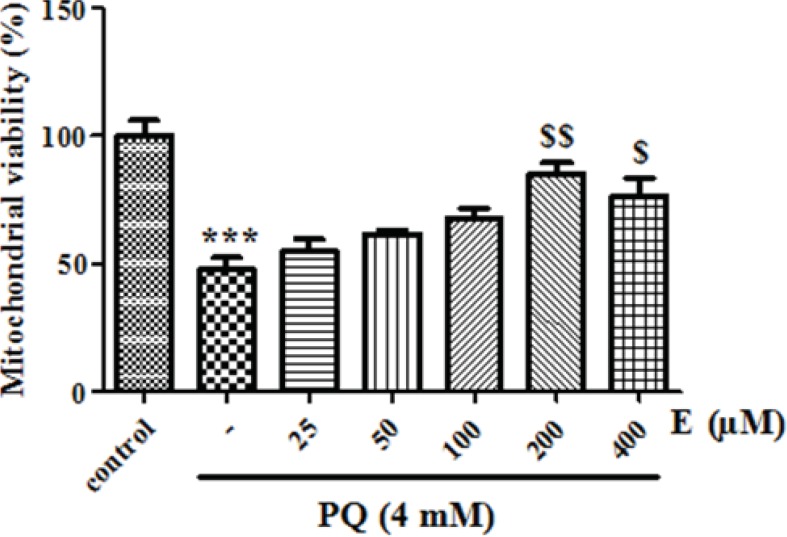
The effect of edaravone on mitochondrial viability

We also examined whether exposure of human lung epithelial A549 cells to PQ would affect mitochondrial viability. As shown in Figure 3, exposure of different concentrations of PQ (1, 2, 4, 6 and 8 mM) for 1 h caused a significant concentration dependent decrease of mitochondrial viability (*P *< 0.05). PQ up to the concentration of 1 mM, did not produce significant mitochondrial toxicity and mitochondrial viability was significantly decreased to 85%, 71%, 50%, 35% and 19% for the concentrations of 1, 2, 4, 6 and 8 μM respectively (p<0.05 for each). Therefore, PQ concentration of 4 mM PQ was selected as LC50 of PQ in isolated lung mitochondria. In addition, edaravone pretreatment (5–100 µM) significantly increased the mitochondrial viability and 200 µM edaravone showed maximal protective effect in isolated lung mitochondria exposed to PQ and was chosen in subsequent mitochondrial experiments (Figure 4). The potential protective effect of edaravone against PQ-induced oxidative stress was assessed by measuring the ROS production in human lung epithelial A549 cells following 12, 24 and 48 h incubation. Table 1 shows that the PQ significantly induced the intracellular production of ROS (p<0.05) and edaravone pretreatment effectively prevented the ROS production induced by PQ (Table 1).As seen in Table 2, PQ induced mitochondrial ROS production had no significant change until 20 min and after that markedly induced ROS production in isolated lung mitochondria as compared to control mitochondria. Whereas, pretreatment with edaravone significantly (p<0.05) prevented the PQ induced mitochondrial ROS production (Table 2).

Isolated lung mitochondria (1 mg protein/mL) were incubated at 37 ^◦^C for 1 h following the addition of different concentration of PQ (0, 1, 2, 4, 6 and 8 mM), mitochondria viability was assessed using succinate dehydrogenase activity assays as described in methods and materials. Data represent the mean±S.D. of six separate experiments.

*P<0.05; compared with control group.

Isolated lung mitochondria (1 mg protein/mL) were incubated at 37 ^◦^C with 25–400 µM edaravone for 5 min and followed by exposure to 4mM PQ for 1 h. mitochondrial viability was assessed using succinate dehydrogenase activity assays as described in methods and materials. Data represent the mean±S.D. of six separate experiments.

***P<0.001; compared with control group.

^$^ *P*<0.05; compared with PQ group.

^$$^ *P*<0.01; compared with PQ group.

**Table 1:** PreventingPQ induced ROS formation by edaravone in human lung epithelial A549 cells.

	**ROS formation (** **Flourescence Intensity)**		
	**12 h**	**24 h**	**48 h**
control	234.7±14.5	253±11	302.3±17
P (500 µM)	450.7±29**	685.7±27.5***	814±37.8***
E (50 µM)	220.7±10	233.7±5.6	278.6±25.5
P (500 µM)+E (50 µM)	301.4±11.2$$$	374.3±20.7$$$	453±23.2$$$

Human lung epithelial A549 cells (10^6^ cells/mL) were incubated at 37 ^◦^C with 50 µM edaravone for 1 h and followed by exposure to 500 µM PQ. At intervals 12, 24 and 48 h after PQ addition, ROS formation was determined through flourometry using DCFH-DA as described in Materials and methods. Data represent the mean±S.D. of six separate experiments.

** *P*<0.001; compared with control group.

*** *P*<0.001; compared with control group.

$$$ *P*<0.001; compared with PQ group.

**Table 2:** PreventingPQ induced ROS formation by edaravone in isolated lung mitochondria

	**ROS formation (** **Flourescence Intensity)**				
	**5min**	**15min**	**30min**	**45min**	**60min**
control	20.2±6	36.9±3.7	51.6±5.5	62±5.6	80.3±13.5
P(4 mM)	29.7±4	61.69	106±5**	195.7±9.6***	299.3±16***
E (200 µM)	19.4±2.4	34.4±3	45.3±6	59±3.6	74.3±12
P(4 mM)+E (200 µM)	21.8±2.8	47.5±3.4$$	70±17$$	93.3±8$$$	104.3±12$$$

Isolated lung mitochondria (1mg protein/mL) were pre-treated at 37 ^◦^C with 200 µM edaravone followed by exposure to 4 mM PQ. At intervals 5, 15, 30, 45 and 60 min after PQ addition, ROS formation was determined through flourometry using DCFH-DA as described in Materials and methods.Data represent the mean±S.D. of six separate experiments.

** *P*<0.001; compared with control group.

*** *P*<0.001; compared with control group.

$$ *P*<0.01; compared with PQ group.

$$$ *P*<0.001; compared with PQ group.

## Discussion

These results clearly revealed that PQ caused oxidative stress in human lung epithelial A549 cells and isolated lung mitochondria. Our data supports the protective effects of edaravone in prevention of PQ cytotoxicity and ROS production in 549 cells. On the other hand, PQ induced mitochondrial ROS production and loss of mitochondrial function significantly inhibited by edaravone.

**Figure 5:**
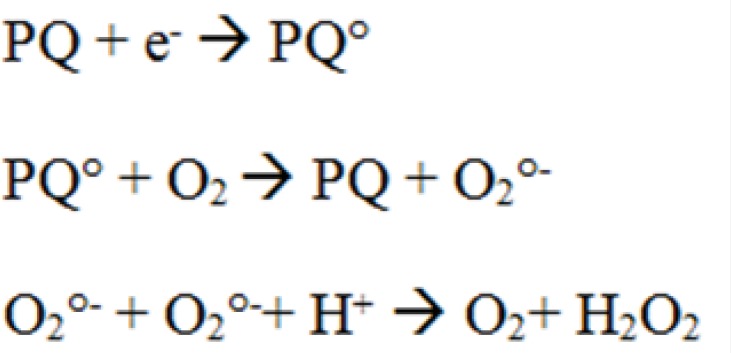
Proposed mechanism for H_2_O_2_ production by paraquat (PQ).

Paraquat is known to have redox-cycling activity whose proposed mechanism of redox cycling for PQ is the enzymatic reduction of PQ to cationic radical (Figure 5) which then can reduce molecular oxygen (O_2_) to superoxide radical (O_2_°^-^) while also regenerating the parent compound (17). Previous studies indicated that mitochondria might be a major cellular component involved in ROS generation induced by PQ (17). Mitochondria are considered as a major source of intracellular ROS such as superoxide (O2^°^^−^) (18, 19). ROS are considered inevitable by-products of mitochondrial respiration resulting from univalent reduction of O_2_ by electrons that leak from the electron transport chain (18). On the other hand, not only mitochondria are the main source of ROS in cell, but they can also be the primary target for ROS that initiate mitochondrial oxidative injury, leading to cell death via apoptosis or necrosis (14).

Our study confirmed outcomes of previous studies, since generation of ROS was significantly increased following PQ treatment in human lung epithelial A549 cells and lung isolated mitochondria as time dependent manner (p < 0.05). Furthermore, results also showed that PQ target lung isolated mitochondria and significantly reduced mitochondrial viability (p < 0.05). Therefore, the ideal drug therapies for alleviating PQ toxicity can be modulating mitochondrial functions by reducing ROS production and inhibiting mitochondrial oxidative damage. Edaravone is a drug that has been used in the treatment of acute ischemic stroke and able to scavenging ROS oxidative, prevent lipid peroxidation and disruption of mitochondrial membranes, thereby can be considered a protective agent for reduction of free radical-mediated injury in several organs (20).On this basis, we evaluated the protective effects of edaravone against PQ toxicity in different experiment models (A549 cells and lung mitochondria). According to our data, edaravone prevented the ROS generation induced by PQ in A549 cells. It also protected A549 cells against oxidative stress damage and decreased the cytotoxicity of PQ. Previous studies suggested that therapeutic effect of edaravone might be related to its antioxidant property and protection of mitochondrial function (2-5). 

It has already been shown that ROS production could cause oxidative damage to the cells, trigger cell death leading to several pathological conditions (21, 22). It has been shown that edaravone decreases free radical-mediated lipid peroxidation *in-vitro* (23). Edaravone also attenuates cerebral edema and tissue injury after recirculation following ischemia in rats (24). Furthermore, edaravone protected PC12 cells from apoptosis and mitochondria damage after oxygen-glucose deprivation-reperfusion injury regulated Bcl2/Bax protein imbalance expression after oxygen-glucose deprivation -reperfusion (4). In addition, Edaravone improved viability of mitochondria that was damaged by PQ, and also decreased PQ-induced ROS generation in mitochondria isolated from rat lung that are in accordance with previous studies. These data indicated that PQ induced cytotoxicity is mediated through oxidative damage to mitochondria which leads to disruption of normal function of mitochondria and ROS generation. Mitochondrial ROS could trigger death pathway in cell which finally results in toxic damage and cell death. Therefore, edaravone as a ROS scavenger, via improving oxidant/antioxidant balance, could inhibit cell death and tissue damage. This effect of edaravone was used for the treatment of clinical situation that mediated oxidative damage to brain and other vital tissue (1, 3 and 5).

Taken together, our study showed that edaravone could be considered a highly promising agent in protecting against PQ-induced oxidative stress damage. As a conclusion, considering its low toxicity our results support the use of edaravone to treat conditions in which PQ toxicity maybe present.

## References

[1] Jiao L, Zhang J, Li Z, Liu H, Chen Y, Xu S (2011). Edaravone alleviates delayed neuronal death and long-dated cognitive dysfunction of hippocampus after transient focal ischemia in wistar rat brains. Neuroscience.

[2] Higashi Y, Jitsuiki D, Chayama K, Yoshizumi M (2006). Edaravone (3-Methyl-1-Phenyl-2-Pyrazolin-5-one), A novel free radical scavenger, for treatment of cardiovascular diseases. Recent Patents on Cardiovascul. Drug Discov.

[3] Okatani Y, Wakatsuki A, Enzan H, Miyahara Y (2003). Edaravone protects against ischemia/reperfusion-induced oxidative damage to mitochondria in rat liver. Europ. J. Pharmacol.

[4] Ying S, Meng L, Ji-cheng L, Er-qing W (2006). Edaravone protects PC12 cells from ischemic-like injury via attenuating the damage to mitochondria. J. Zhejiang Univ. SCIENCE B.

[5] Takayasu Y, Nakaki J, Kawasaki T, Koda K, Ago Y, Baba A, Matsuda T (2007). Edaravone, a Radical Scavenger, Inhibits Mitochondrial Permeability Transition Pore in Rat Brain. J. Pharmacol. Sci.

[6] Pourahmad J, Hosseini M-J, Bakan S, Ghazi-Khansari M (2011). Hepatoprotective activity of angiotensin-converting enzyme (ACE) inhibitors, captopril and enalapril, against paraquat toxicity. Pesticide Biochem. Physiol.

[7] Sabzghabaee AM, Eizadi-Mood N, Montazeri K, Yaraghi A, Golabi M (2010). Fatality in paraquat poisoning. Singapore Med. J.

[8] Zhi Q-M, Yang L-t, Sun H-c (2011). Protective Effect of Ambroxol against Paraquat-induced Pulmonary Fibrosis in Rats. Intern. Med.

[9] Ghazi-khansari M, Mohammadi-Bardbori A, Hosseini M-J (2006). Using janus green B to study paraquat toxicity in rat liver mitochondria role of ACE inhibitors (Thiol and Nonthiol ACEi). Annals New York Acad. Sci.

[10] Lopez E, Arce C, Oset-Gasque MJ, Canadas S, Gonzalez MP (2006). Cadmium induces reactive oxygen species generation and lipid peroxidation in cortical neurons in culture. Free Radical. Bio. Med.

[11] Pourahmad J, Eskandari MR, Alavian G, Shaki F (2011). Lysosomal membrane leakiness and metabolic biomethylation play key roles in methyl tertiary butyl etherinduced toxicity and detoxification. Toxicological. Environmental. Chem.

[12] Moreira PI, Custódio JBA, Nunes E, Oliveira PJ, Moreno A, Seic R, Oliveira CR, Santos MS (2011). Mitochondria from distinct tissues are differently affected by 17B-estradiol and tamoxifen. J. Steroid Biochem. Molecular Biol.

[13] Pourahmad J, Eskandari MR, Kaghazi A, Shaki F, Shahraki J, Fard JK (2012). A new approach on valproic acid induced hepatotoxicity: Involvement of lysosomal membrane leakiness and cellular proteolysis. Toxicol. In-vitro.

[14] Shaki F, Hosseini M-J, Ghazi-khansari M, Pourahmad J (2012). Toxicity of depleted uranium on isolated rat kidney mitochondria. Biochim. Biophys. Acta.

[15] Bradford MM (1976). A rapid and sensitive method for the quantitation of microgram quantities of protein utilizing the principle of protein-dye binding. Anal. Biochem.

[16] Shaki F, Hosseini M-J, Ghazi-khansari M, Pourahmad J (2013). Toxicity of Arsenic (III) on Isolated Liver Mitochondria: A New Mechanistic Approach. Iran. J. Pharm. Res.

[17] Castello PR, Drechsel DA, Patel M (2007). Mitochondria Are a Major Source of Paraquat-induced Reactive Oxygen Species Production in the Brain. J. biol. Chem.

[18] Chance B, Sies H, Boveris A (1979). Hydroperoxide metabolism in mammalian organs. Physiol. Rev.

[19] Miwa S, St-Pierre J, Partridge L, Brand MD (2003). Superoxide and hydrogen peroxide production in Drosophila mitochondria. Free Radical Bio. Med.

[20] Qiu W, Gu H, Zheng L, Zhou J, Chen D, Chen Y (2008). Pretreatment with edaravone reduces lung mitochondrial damage in an infant rabbit ischemia-reperfusion model. J. Pediatric Surgery.

[21] Hosseini M-J, Shaki F, Ghazi-khansari M, Pourahmad J (2013). Toxicity of arsenic (III) on isolated liver mitochondria: a new mechanistic approach. Iran. J. Pharm. Res.

[22] Houot V, Etienne P, Petitot A, Barbier S, Blein J, Suty L (2001). Hydrogen peroxide induces programmed cell death features in cultured tobacco BY-2 cells, in a dose-dependent manner. J. Exp. Bot.

[23] Watanabe T, Yuki S, Egawa M, Nishi H (1994). Protective effects of MCI-186 on cerebral ischemia: possible involvement of free radical scavenging and antioxidant actions. J. Pharmacol. Exp. Ther.

[24] Abe K, Kogure K (1988). Strong attenuation of ischemic and postischemic brain edema in rats by a novel free radical scavenger. Stroke.

